# Contextual connectivity: A framework for understanding the intrinsic dynamic architecture of large-scale functional brain networks

**DOI:** 10.1038/s41598-017-06866-w

**Published:** 2017-07-26

**Authors:** Rastko Ciric, Jason S. Nomi, Lucina Q. Uddin, Ajay B. Satpute

**Affiliations:** 1 Dept. of Neuroscience, Pomona College, Claremont, CA USA; 2 Dept. of Psychology, University of Miami, Coral Gables, FL USA; 3 Dept. of Psychology, Pomona College, Claremont, CA USA

## Abstract

Investigations of the human brain’s connectomic architecture have produced two alternative models: one describes the brain’s spatial structure in terms of static localized networks, and the other describes the brain’s temporal structure in terms of dynamic whole-brain states. Here, we used tools from connectivity dynamics to develop a synthesis that bridges these models. Using resting fMRI data, we investigated the assumptions undergirding current models of the human connectome. Consistent with state-based models, our results suggest that static localized networks are superordinate approximations of underlying dynamic states. Furthermore, each of these localized, dynamic connectivity states is associated with global changes in the whole-brain functional connectome. By nesting localized dynamic connectivity states within their whole-brain contexts, we demonstrate the relative temporal independence of brain networks. Our assay for functional autonomy of coordinated neural systems is broadly applicable, and our findings provide evidence of structure in temporal state dynamics that complements the well-described static spatial organization of the brain.

## Introduction

A major endeavor in neuroscience is to characterize the spatiotemporal organization of the brain into functional systems^1^. By identifying patterns of synchronous brain activity, functional magnetic resonance neuroimaging (fMRI) techniques have partitioned the human brain into large-scale networks^2, 3^. These functional networks are stable across individuals and populations^4–6^, are roughly consistent across task-evoked and resting data^7, 8^, and are present across mammalian species^9, 10^. A hierarchically modularized set of canonical networks is now widely accepted as an organizational principle of the brain^11, 12^. Indeed, an expanding literature relates networks to specific psychological functions and individual differences^13–16^, with the potential for improved clinical diagnosis or treatment outcome metrics^17–19^.

However, a growing body of work has called into question how accurately this canonical network model represents underlying neural architecture. In particular, many methods used to delineate networks rely on two implicit assumptions. First is the ‘spatial assumption’ that each brain region participates in exactly one network. Casting doubt on this are models suggesting that brain regions can engage with several different networks^20–26^, dynamic causal models showing that connectivity between brain regions changes as a function of the experimental context^27^, and graph theoretic models intimating the existence of neural hubs that recruit multiple networks^28–31^. Second is the ‘temporal assumption’ that the connectivity within each network remains relatively stable over time. This, too, has been called into question, with recent work^32^ suggesting that the brain is dynamically multistable. That is, the brain may occupy any of a number of connectivity states over time, each with a distinct network architecture^33^. Such a multistable model has been applied to discover novel biomarkers for pathology^34–36^ and to track changing cognitive demands^37^.

While the idea that brain connectivity reconfigures over short periods of time is gaining momentum in the dynamic functional connectivity literature^33, 38^, investigations of dynamic connectivity are often couched in the vocabulary of ‘brain states’. This language is not trivially relatable to the more commonplace terminology of brain networks: brain states span the entire brain and exist transiently; by contrast, brain networks are more often described as spatially localized but temporally persistent, or static. If the brain can be modelled as a series of non-localized states, where does that leave brain networks? Recent work has gone to some lengths toward a synthesis of static network- and dynamic brain state-based models. For instance, transient patterns of connectivity have been observed among nodes in localized networks^25, 39, 40^. While these studies examined states within one or another network, recent work further suggests that multiple states occur in many of the major networks, that these states provide information about states in other networks, and that state transitions may relate to individual differences in behavior (i.e. schizophrenia)^41^. Such findings highlight the importance of analytical approaches that capture a balance between the utility offered by both state- and network-based models. Here, we make a contribution toward the state-network synthesis. Like concurrent work^41^, we use state-based models to investigate the information exchange among brain networks; however, we evaluate the hypothesis that brain networks are relatively independent, thus providing a novel conceptualization of the relation between brain states and brain networks.

**Figure 1:**
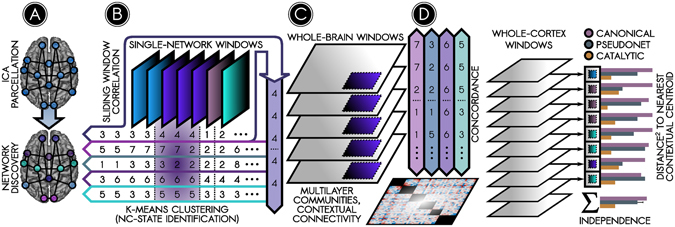
Summary of analysis steps. (**A**) ICA was used to parcellate the human brain into 70 cortical and 10 subcortical/cerebellar nodes (top). Cortical nodes were assigned to six canonical functional networks using a community detection algorithm (bottom; results in Fig. 2). (**B**) Temporal fluctuations in connection strength between nodes were identified using sliding-window correlations. A k-means clustering analysis identified prominent, recurring ‘network connectivity states’ (NC-states) among nodes within each canonical network. The top row schematically illustrates four recurring NC-states of one canonical network. The bottom rows show the NC-states in other networks for corresponding time windows (results in Fig. 3A). To see how NC-states in a given network relate to the connectivity state of the rest of the brain, two approaches were taken. One approach averaged the connectivity matrices over a given NC-state’s time windows (such as for NC-state 4 for the network in the top row in the schematic) to determine (**C**) the whole-brain connectivity context (WBCC, grey portion of matrix) in which each NC-state (purple portion) occurred (results in Fig. 3B). The other approach examined synchrony between NC-states in different canonical networks using a Bayesian concordance matrix (**D**) *bottom*) to test whether NC-states in different networks *(top)* relate to one another over time (results in Supplementary Figure 4).

In the present study, we evaluate the stability, homogeneity, and independence of six networks to determine whether each network’s temporal variability constitutes stochastic variation about its time-averaged structure, or whether it reflects temporally distinct network connectivity states (or NC-states). We identify putative NC-states using k-means clustering of connections computed over a sliding window^32^ (Fig. 1). We next characterize the whole-brain milieu in which NC-states occur, identifying novel organizational principles from which to further understanding of the brain’s intrinsic architecture. Our findings diverge from assumptions of spatiotemporal stability, and suggest instead that canonical networks are superordinate representations of several NC-states, each of which describes the state of a network at a given moment in time and is associated with a distinct whole-brain context. Surprisingly, we also find that individual NC-state connectivity is relatively independent of global (whole-cortex) connectivity. Based on these findings, we develop a novel organizational framework, *contextual connectivity*, towards reconciling network- and state-based models of the human brain.

## Results

### Canonical network identification

**Figure 2:**
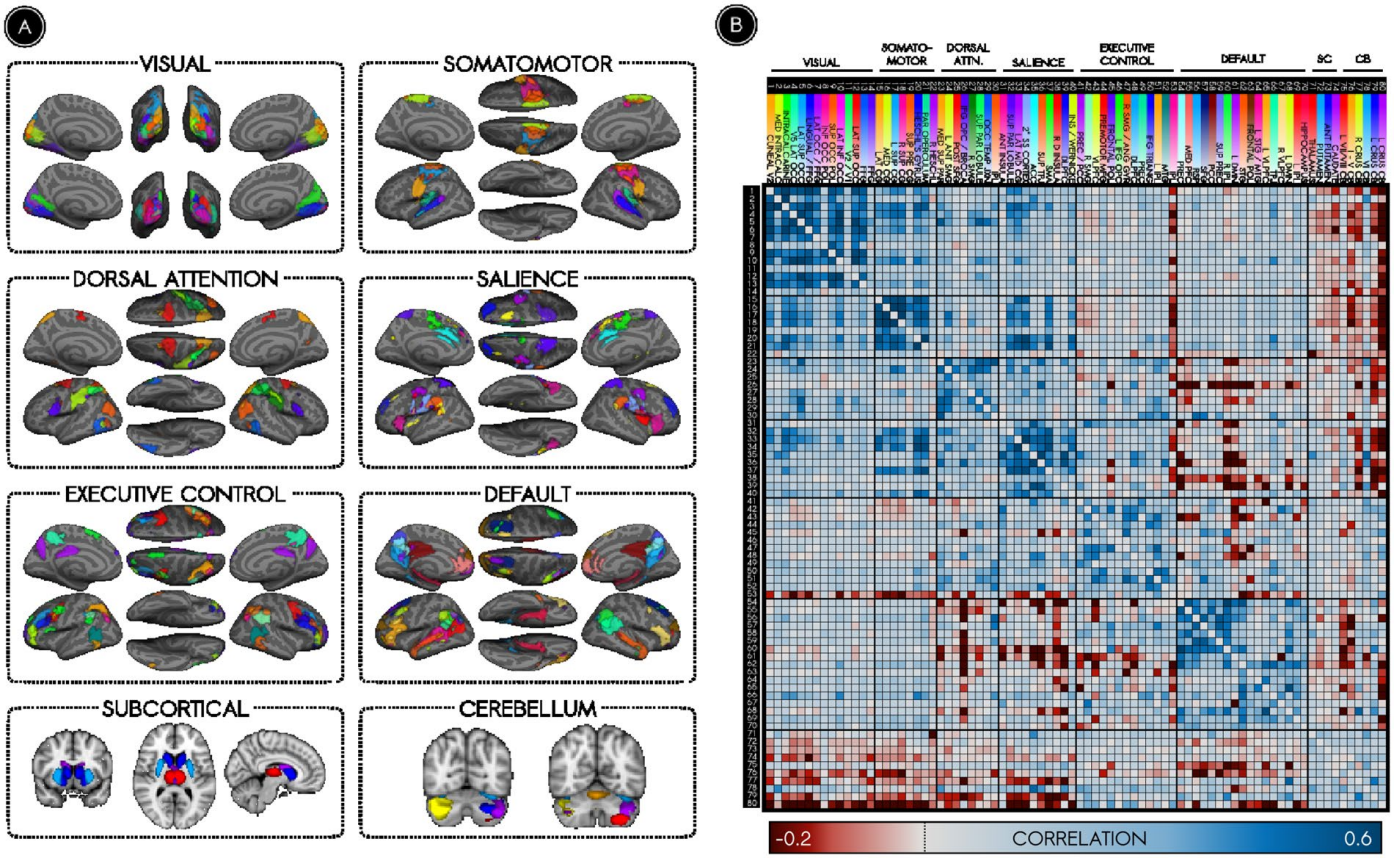
Canonical functional networks of the time-averaged connectome. (**A**) Spatial maps of nodes are illustrated according to their membership in functional networks. (**B**) Strengths of connections between nodes are illustrated in a whole-brain adjacency matrix. The strongest connections of the time-averaged functional connectome occur among nodes of the same network.

The spatial segregation of cortical parcels into functional brain networks forms the basis of modern analyses of the human connectome. Here, we reproduced this canonical network partition as the first step toward a revised understanding of the relation between static networks and dynamic brain states. Specifically, we used spatial independent component analysis (ICA)^42^ to localize 80 sources of non-noise variance in the BOLD signal. A connectome was defined using the 80 components as nodes and using the Pearson correlation coefficients between node timeseries as evidence of connection strength^43^. Nodes were assigned membership to canonical brain networks by training a community detection algorithm^44, 45^ using an *a priori* hypothesis^3, 46^. We thus obtained six communities of nodes exhibiting one-to-one correspondence with six reference brain networks: the visual network (VIS), the somatomotor network (SOM), the dorsal attention network (DAT), the cingulo-opercular/salience network (SAL), the executive control network (EXE), and the default mode network (DMN)^2, 3^. Spatial cross-correlations between these communities and reference networks ranged from approximately 0.49 to 0.68, well above a previously established cutoff^46^, a positive indication that the canonical partition was reproduced (Supplementary Figure 1C). Figure 2A illustrates the clear spatial correspondence between the networks of our partition and canonical functional networks^2, 3, 32^. Figure 2B depicts the time-averaged static network connectome; the majority of strong and specific correlations among nodes were localized to within-network connections. We used this partition to organize subsequent analyses examining the moment-to-moment connectivity of functional networks.

### Each canonical network is resolvable into a set of network connectivity states (NC-states)

**Figure 3:**
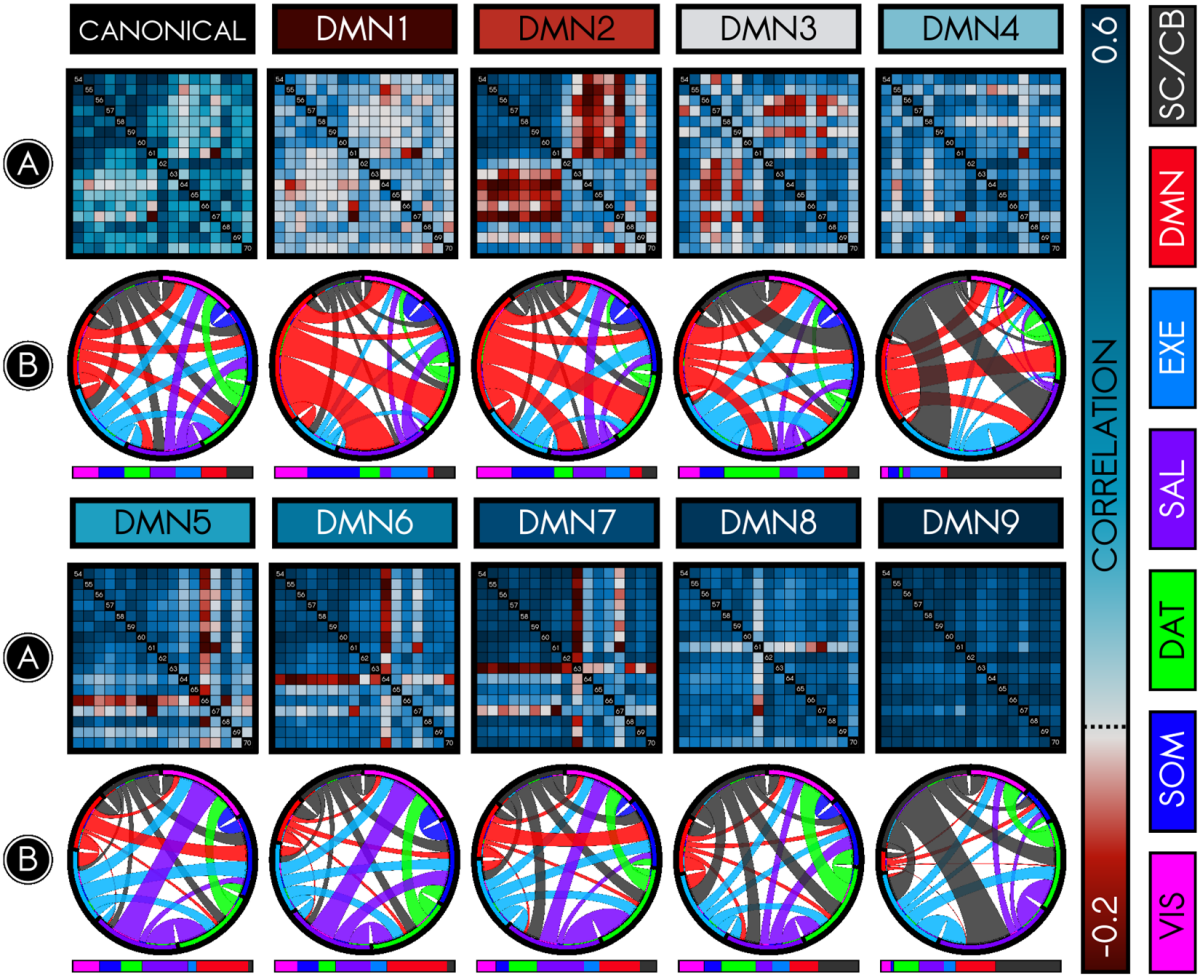
Connectivity dynamics of the default mode network (DMN). Dynamic functional connectivity analysis among nodes of the DMN revealed that the DMN is composed of several network connectivity states (NC-states). This figure summarizes the connectivity patterns of the canonical (time-averaged) DMN (*first row*, *left*) in comparison with the 9 observed NC-states (DMN1–9), organized in order of low intrinsic connectivity (dark red) to high intrinsic connectivity (dark blue). (**A**) The connectivity (Pearson correlation) among the 17 nodes in the DMN, either time-averaged or for specific NC-states. Each NC-state exhibits a distinct profile of connectivity among nodes. (**B**) Relative within- and between-network allegiance for each NC-state and its whole-brain context. Allegiance is the probability that nodes are in the same community when that NC-state is present. The chord diagram illustrates *between-network* allegiances for each NC-state relative to the time-averaged connectome. The color codes along the rim signify different canonical networks as labeled at right (e.g., red for DMN). Longer rim segments indicate that the network has greater allegiance to other networks relative to the time-averaged state, and the size of connections between rim segments indicate strength of between-network allegiances. The time-averaged chord diagram defines a ‘baseline’; thus, each rim segment is of equal size. The remaining plots show that allegiance between networks changes across NC-states (to emphasize changes in allegiance, plotted allegiance ratios were rescaled exponentially). The adjacent bar illustrates *within-network* allegiance using corresponding color codes. Longer segments indicate that the network has greater intrinsic allegiance, again relative to the time-averaged ‘baseline’. For instance, DMN in DMN1 exhibits weak within-network allegiance but strong between-network allegiance, which appears to be driven by greater allegiance to DAT, SAL, and VIS. DMN’s intrinsic allegiance increases from DMN1 to DMN9. Conversely, DMN’s allegiance to other networks substantially drops from DMN1 to DMN9.

While the brain’s organization into networks ranks among the most robust findings in functional connectivity, the stability of brain networks during rest is under dispute^32, 47^. Recent evidence suggests that the moment-to-moment synchrony among the regions constituting each functional network may deviate markedly from that network’s canonical structure^32^. Here, we evaluated this hypothesis. For each of the six networks, we calculated the extent to which different ‘network connectivity states’ (NC-states) could arise among nodes in the network. We began by using k-means clustering to identify putative NC-states for each canonical network. Each NC-state represented a distinct pattern of synchrony among the nodes within a network. Compared with expectation under permuted and phase-shifted null models, the NC-states we observed were well-differentiated (*p* < 0.01) and represented distinct clusters (*p* < 0.01; Supplementary Figure 2). Figure 3A illustrates the NC-states of the DMN sorted in ascending order of intrinsic connectivity.

Inconsistent with models that suggest uniformity of brain networks over time, and consistent with recent results using dynamic functional connectivity^41^, we found that networks could be resolved temporally into NC-states. This result parallels and extends the observation that the whole connectome can be resolved temporally into whole-brain states^32^. Remarkably, no NC-state was completely predominant for any network. Instead, all subjects exhibited multiple NC-states per scan; reciprocally, the many NC-states observed per network were not idiosyncratic to particular subjects. For the default mode network, for instance, between 7 and 9 NC-states were represented in the majority of subjects, with no subject exhibiting fewer than 4. Moreover, gross features of NC-states were conserved at the single-subject level, and the majority of NC-states replicated across split-half samples (32 out of 46 NC-states replicated). Taken together, these results indicate that each brain network is incompletely characterized by its time-averaged connectivity profile.

### NC-states occur in specific whole-brain connectivity contexts (WBCCs)

We used NC-states as the building blocks of a framework for evaluating the *temporal independence* of canonical networks. We first posed the question: Given information about the state of one brain network, can we make inferences about the state of another network, or of the brain as a whole? To answer this question, we fashioned hypotheses based on whole-brain connectivity contexts (WBCCs)^48^. A WBCC was defined as *the global connectomic environment associated with specific local changes in the connectome*. We mathematically modelled the WBCC of each NC-state as the average (global connectomic) environment in which that (local) NC-state was present. We used multilayer community detection^45^ to obtain an allegiance matrix ^49^ representing each NC-state’s WBCC. In an allegiance matrix, the weight of the edge connecting a pair of nodes represents the probability that those nodes will be found in the same community over all time windows during which that NC-state is present. Allegiance matrices, unlike correlation matrices, are more sensitive to specificity than to magnitude of connections. We first tested a model of *local-global temporal independence*: the null hypothesis that an NC-state’s WBCC did not significantly differ from the time-averaged connectome, which would suggest that each network’s connectivity state changed independently of the remainder of the brain. In contrast with the null model derived under this hypothesis, all NC-states were associated with specific changes in the functional architecture of the whole brain (see Methods). Figure 3B uses a chord diagram to illustrate changes in the WBCC associated with each NC-state. For example, DMN4 was associated with increased allegiance among salience, executive, and subcortical systems, while DMN7 was associated with increased allegiance between the salience and dorsal attention networks (relative to the time-averaged/static connectome). In supplementary analyses, we also tested interdependence between individual networks in another way by using a Bayesian *concordance* metric. This showed that the occurrence of a state in one network was predictive of the occurrence of particular states in other networks (*p* < 0.05; Supplementary Figure 3). These results are inconsistent with complete local-global temporal independence of brain networks, and instead support the view that information about the state of the entire brain is embedded in each network.

### Dynamically determined NC-states recapitulate time-averaged subnetworks

**Figure 4:**
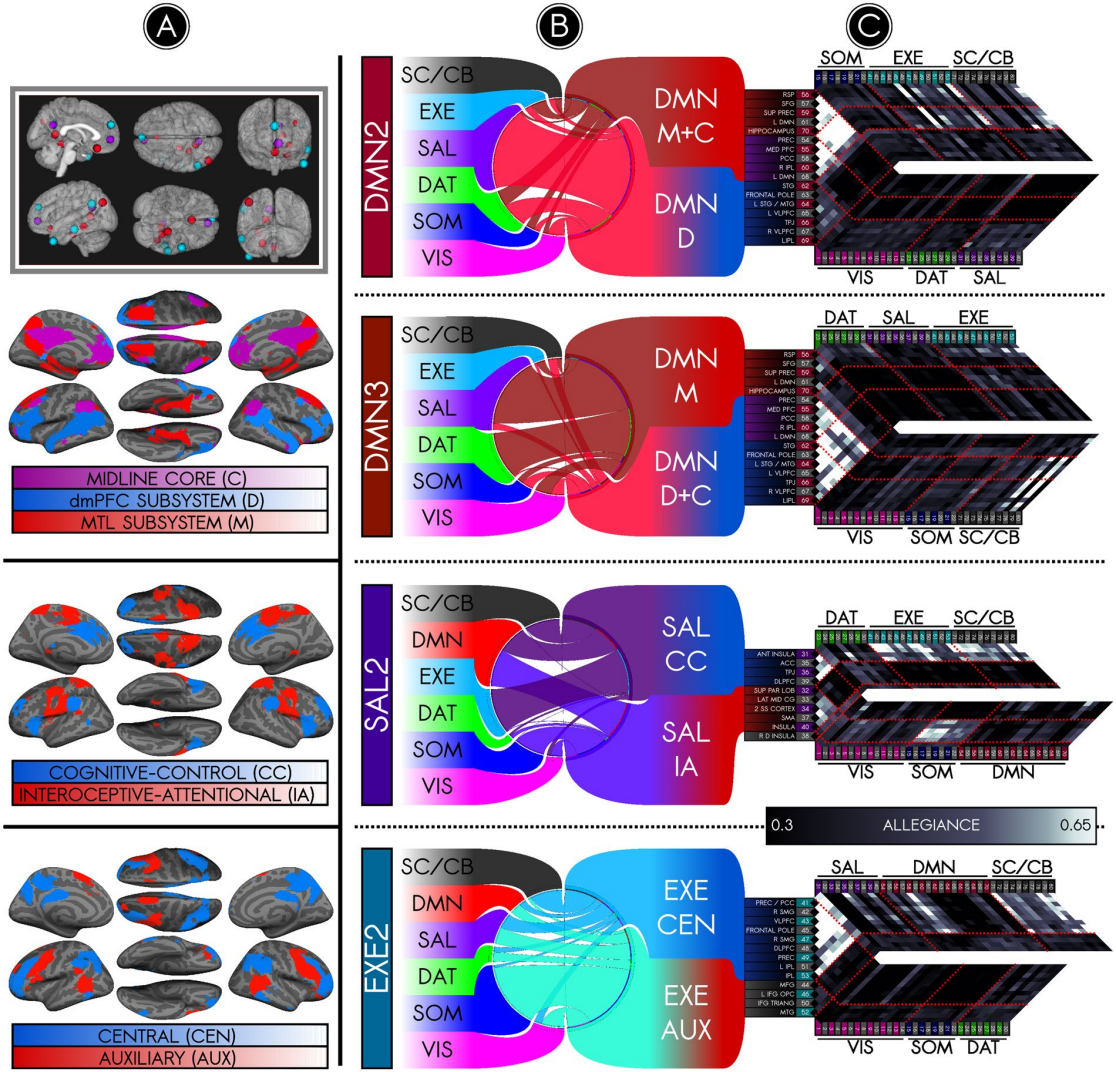
The default mode, salience, and executive networks dynamically segregate into temporally decomposable but spatially overlapping subsystems. *Inset*, *top left*: The inset illustrates subsystems of the DMN from a study of the DMN’s intrinsic structure^15^. We extended these findings, which used time-averaged connectivity, by using our dynamic connectivity approach. Subsystems were identified in NC-states by calculating the allegiance between each network’s nodes and all other nodes, and then clustering the nodal allegiances to examine whether they associated into subsystems. For example, the network connectivity profile for DMN2 was obtained by submitting the 17 (DMN nodes) x 80 (all nodes) allegiance matrix during DMN2, as shown in column (**C**), to a hierarchical clustering analysis, which revealed two types of nodes in DMN2 (cohesive and non-cohesive). The same analysis was performed on DMN3, which also revealed cohesive and non-cohesive nodes. (**A**) Summarizing across both NC-states, the clustering analyses revealed three sets of nodes. The spatial layout of these DMN subgroups are displayed in one figure (*below inset*). One set of nodes (purple) was cohesive (i.e. the nodes clustered together on the basis of allegiance) during both DMN2 and DMN3, and corresponded to the midline core (“C”) from time-averaged studies^15^. Another set of nodes (blue) was cohesive during DMN3 along with C but lacked cohesion during DMN2, and corresponded to a dmPFC subsystem (“D”). A third set of nodes (red) was cohesive during DMN2 along with C but lacked cohesion during DMN3, and corresponded to a MTL subsystem (“M”). These findings recapitulate DMN subnetworks observed in time-averaged connectivity (inset), but on a dynamic level. Thus, our findings suggest that these two subnetworks are engaged during different NC-states, rather than contemporaneously. (**B**,**C**) Our analysis also enables examining how these subnetworks relate to other networks in the brain. During DMN2, nodes in the M + C subsystem modularized relative to the time-averaged connectivity profile, as illustrated in the chord diagram by narrower chords extending from the DMN M + C to other networks, and as also illustrated in the allegiance matrix. Non-cohesive nodes in DMN D showed reduced modularization. In contrast, during DMN3, nodes in the D + C subsystem modularized relative to the time-averaged connectivity profile, and non-cohesive nodes in DMN M showed reduced modularization. *Rows 3 and 4*: We applied this same analysis to other networks. Two other NC-states, SAL2 and EXE2, also evinced bifurcation into subnetworks. SAL2 was characterized by a bifurcation of the salience network into two sets of nodes, each exhibiting distinct and antagonistic connectivity profiles as shown by the chord diagrams and network connectivity profiles (see Supplementary Figure 3 for clustering trees). One set of SAL nodes (blue) referred to as the cognitive-control core (CC) joined the EXE network. Another set of nodes (red) referred to as the interoceptive periphery (IA) joined the SOM network. The fourth row shows findings for EXE2, which was characterized by enhanced modularization of a central executive system (CEN, blue) and demodularization of auxiliary nodes (AUX, red).

The analyses above suggest that brain networks are temporally resolvable into transient NC-states. However, it is not yet clear that this added complexity also adds notable value. To address this, we first examined whether the NC-states we observed using dynamic analyses recapitulate prior results from static connectivity analyses. Such work has shown that the DMN is composed of two subnetworks, one anchored in the medial temporal lobe (MTL) and the other in the dorsomedial prefrontal cortex (DMPFC), both of which converge onto a ‘midline core’ (Fig. 4A, inset). We first examined whether our analysis reproduced these subnetworks of the DMN. We used hierarchical clustering to determine whether a network’s nodes associated into subnetworks (Supplementary Figure 4). NC-states DMN2 and DMN3 showed a clear correspondence with previously reported MTL and DMPFC subnetworks, respectively. DMN2 featured enhanced connections among medial nodes (red and violet in Fig. 4A), while DMN3 featured enhanced connections among lateral nodes (blue and violet). The precuneus, PCC, medial PFC, and right IPL - nodes that remained cohesive in both NC-states (violet) - map onto the midline core of the DMN following prior work^50^ (Fig. 4A, inset). These findings validate our dynamic approach insofar as it captures known findings from static connectivity studies.

We then addressed two additional questions afforded by our dynamic approach. First, what is the status of the DMPFC nodes during DMN2, and of the MTL nodes during DMN3? Our findings indicate that in both cases the nodes in question demodularize (Fig. 4B,C). That is, the connectivity of the DMPFC subnetwork with other DMN nodes diminishes in DMN2, while its connectivity with other networks (e.g. SAL) increases. Similarly, the connectivity of the MTL subnetwork with other DMN nodes decreases in DMN3 while increasing with other networks (e.g. DAT and SAL). Second, we explored whether networks other than DMN also have NC-states that suggest the configuration of nodes into subnetworks. We observed NC-states corresponding to fractionation of the SAL and EXE networks (and replicated these in split-half samples). In SAL2, salience nodes assorted into two antagonistic subsystems (Fig. 4A). We suggest putative functions based on their distinct connectivity profiles, but caution that a definitive functional description would require formal probing using tasks. SAL2 exhibited a ‘cognitive-control’ core (blue) that became negatively correlated with an ‘interoceptive-attentional’ periphery (red). While the ventral core remained selectively connected to EXE, the dorsal periphery connected strongly into SOM and VIS (Fig. 4B,C). As for the EXE network, EXE2 was characterized by a splitting of the EXE into two sets of nodes (Fig. 4B,C). While one set of nodes appeared to lose cohesion with the rest of EXE and with one another (red), another set of nodes exhibited greater modularization (blue). The DMN subsystems coherent in DMN2 and DMN3 were similarly modularized (Fig. 4B). These modularizations may reflect a shift toward specific, localized computation in these subsystems.

### Canonical networks are relatively independent

**Figure 5:**
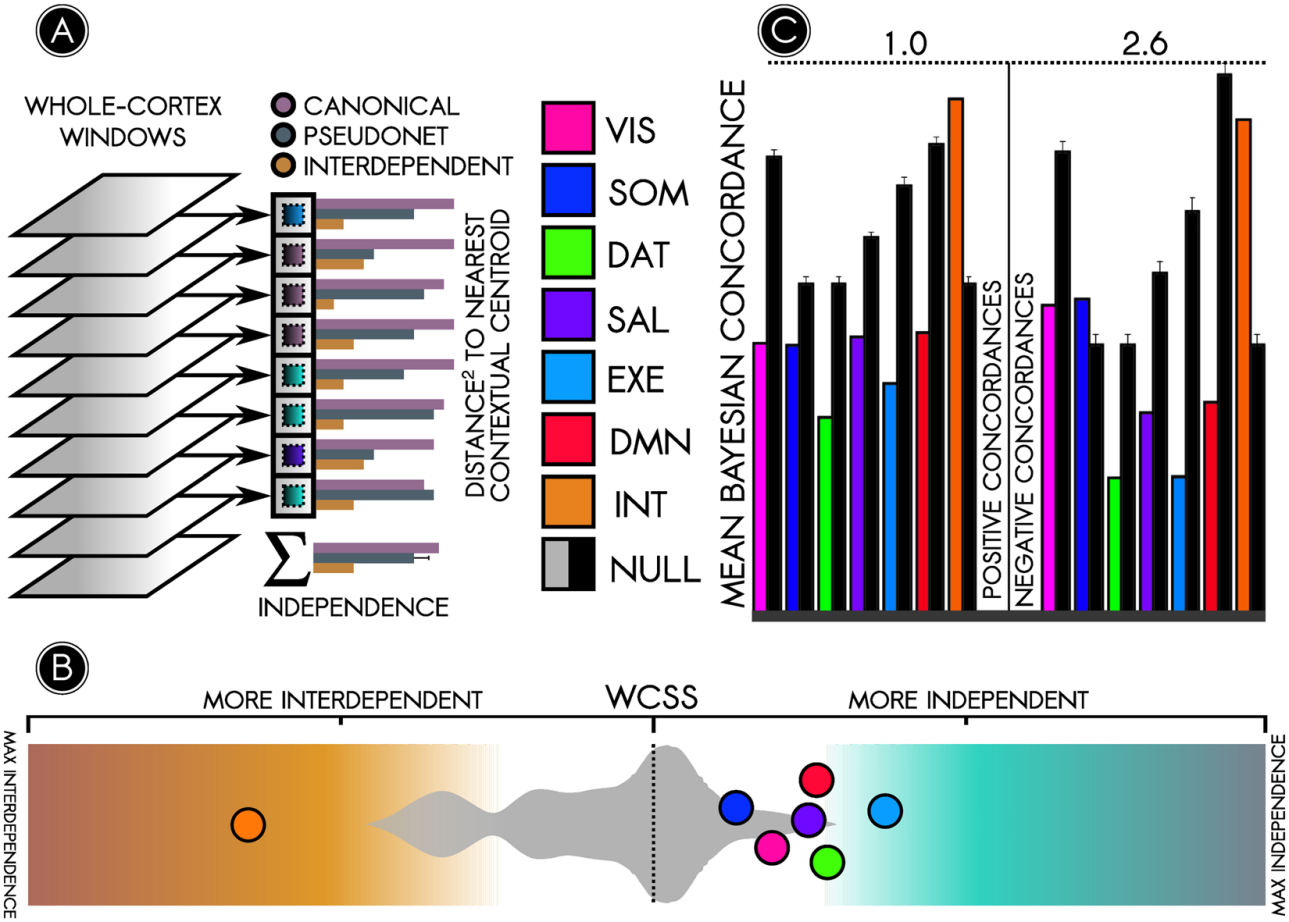
Canonical networks are significantly more temporally independent than random cortical subsystems. The prior analysis suggests that the constitution of brain networks may change over time as parts of those networks decohere or modularise. Given this information, the utility of the canonical network architecture in a dynamic framework is unclear. We tested this by asking the following statistical question: given the dynamic states among a selection of nodes, how well do these dynamic states explain dynamic variations in global (whole-brain) connectivity? If local changes in canonical brain networks (such as the DMN) largely recapitulate global changes across the entire connectome, then canonical networks are perhaps not as useful as whole-brain states as an organizational framework for dynamic connectivity. If, by contrast, the connectivity of canonical brain networks changes in a manner that is relatively independent of the rest of the connectome, then network-level metrics could provide an important complement to global metrics. To assess these possibilities, we examined how well each canonical brain network accounted for dynamic variations in the whole brain, by calculating the within-cluster sum-of-squares error (see methods for details), as illustrated in (**A**). This metric takes the whole-brain connectivity at each time point (grey windows at left), identifies the nearest whole-brain context among a network’s NC-states (depicted by the arrow pointing to the small square, the color of which signifies a different NC-state and the grey boundary its corresponding whole-brain context), and calculates a distance or error measure (indicated by the horizontal bar plot). Summing across all time points provides a measure of how well a canonical network – given its NC-states – explains variation in the whole brain, or conversely, how informationally independent the network is – given its NC-states – from the rest of the brain. A null distribution was built by applying the same approach to random pseudo-networks. (**B**) The WCSS approach demonstrated that canonical networks (colored circles) were more independent than random pseudo-networks (grey distribution plot, background) (*p* < 0.05 for all except SOM, *p* = 0.05 for SOM). (**C**) A separate metric, the mean Bayesian concordance, recapitulated the results obtained using the WCSS error. Here, greater concordance corresponds to more interdependence. Each canonical network (colored bars) was compared against a null distribution of pseudo-networks (black bars). With one exception, canonical brain networks were significantly more independent (*p* < 0.01).

The preceding analyses provide evidence of interdependence among brain networks but do not test whether networks nonetheless retain a relative degree of independence. Indeed, it is notable that temporal independence of networks has largely been assumed on the basis of community structure rather than formally tested. We reasoned that for a given sample of nodes, the extent to which these nodes’ connections cannot explain dynamic connectivity among the remaining nodes is the extent to which these nodes are *independent*. Owing to the putative modularization of canonical brain networks, we hypothesized that dynamics of known networks would poorly explain the entire cortex’s dynamics in comparison with dynamics of a random sample of nodes, or a *pseudo-network*. We calculated how well the NC-states of canonical and pseudo-networks explained the temporal variation in the cortical connectome using the within-cluster sum of squares (WCSS) error metric. The WCSS error is a measure of the quality of a putative clustering solution, or the extent to which it explains the variability within a dataset; a higher WCSS error corresponds to a poor-quality clustering solution, which may be interpreted as evidence that a (pseudo-) network’s local connections poorly predict global connectivity; i.e., the (pseudo-) network is more independent. We proposed whole-cortex clustering solutions on the basis of only information from connections within each (pseudo-)network and calculated the WCSS error for each proposed solution. Consistent with our hypothesis, dynamics of canonical networks explained significantly less whole-cortex variation than did dynamics of pseudo-networks (Fig. 5A; *p* < 0.05 except *p* = 0.05 for SOM). Furthermore, canonical network NC-states were significantly less concordant with cortical states than were pseudo-network NC-states (Fig. 5B; *p* < 0.01 except SOM, permutation test). These findings suggest that, while fluctuations occurring on a dynamic level across networks are of importance^41^, canonical networks nevertheless retain a degree of independence from the rest of the cortex.

**Figure 6:**
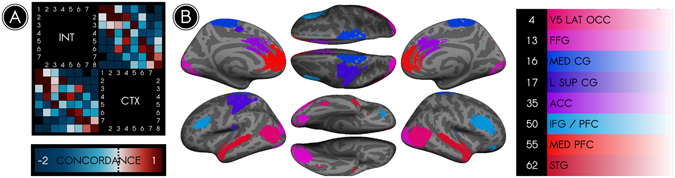
The most interdependent brain system consists primarily of connections between nodes in different networks. We identified an interdependent (INT) node set characterized by its high temporal interdependence with the cortex as a whole (CTX). (**A**) The Bayesian concordance between states of INT and states of CTX is represented as a state-by-state matrix; a positive concordance indicates that states co-occur more often than predicted by chance. Every state of INT was highly concordant (*concordance* > 1) with at least one state of CTX, reflective of similarities between the trajectories of INT and CTX through their respective state spaces. (**B**) The 8 nodes in the INT set, color coded according to their parent canonical network and listed at right. These nodes differed from graph-theoretical hubs; rather than being situated in areas where multiple networks overlapped, they were typically representative nodes of their parent networks. Because these nodes represented a diversity of brain networks, nearly all connections of INT were *between-network* rather than *within-network* connections.

As an exploratory aim, we used these measurements to characterize a maximally interdependent brain subsystem. We hypothesized that such a ‘hub’ system would better explain cortical dynamics than would pseudo-networks and that it would exhibit NC-states highly concordant with cortical states. Several subsystems satisfied these criteria; of these, the most potent was the ‘most interdependent’ (INT) subsystem presented in Fig. 6 (*p* < 0.01 for WCSS and concordance metrics). INT nodes included medial and lateral prefrontal cortices, midcingulate gyrus, middle temporal gyrus, fusiform gyrus, dorsal somatomotor cortex, and lateral occipital cortex. Unlike prior attempts to identify hubs, INT nodes were not characterized by high degree^18^ or participation coefficient^28^. Instead, they appear to be representative nodes of their parent networks and may lack substantial anatomical connections with one another. It is possible that traditional graph-based hubs entrain synchrony among INT nodes.

## Discussion

An ideal model of the brain distills its dynamic, high-dimensional information^27^ into interpretable constructs without sacrificing fidelity. Towards this goal, research using functional connectivity has centered on two models. The first of these models emphasizes the spatial dimension, and parcellates brain activity into spatially localized static functional networks^2, 3, 5^. A second model emphasizes the temporal dimension, and parcellates brain activity into dynamically recurring states^32, 35, 36, 51^. While both network and state models are supported by an extensive literature, it remains unclear how dynamic brain states and static brain networks relate. Here we contribute to this literature by (i) demonstrating that the brain’s dynamic structure is consistent with its spatial structure (i.e. the most independent systems of the brain, even when conducting dynamic analyses, remain the major networks) and (ii) demonstrating that brain networks’ local states change in a manner that is relatively independent of the global brain state.

In the present study, we deconstructed the elementary units of both models – the spatial network and the temporal state – into a ‘common factor’, or a spatially localized connectivity state (i.e., a NC-state). We then evaluated the assumptions underlying naive network-based models, in particular the assumptions that brain networks are independent and stable. Inconsistent with the canonical model of localized and temporally stable networks (and consistent with the dynamic connectivity literature)^32, 41^, we found that canonical brain networks are temporally decomposable into an array of possible connectivity states. Moreover, each local connectivity state also provides information about the global state of the entire brain. However, inconsistent with whole-brain models that disregard network boundaries, our results also indicate that networks retain a degree of modularization; connectivity patterns among nodes of a single network provide less information about the whole-brain dynamic state than do connectivity patterns among randomly selected nodes.

To accommodate these findings, we advance an alternative model, *contextual connectivity*, and a corresponding analytical framework. In our model, networks are better thought of as composed from a set of dynamically recurring NC-states, each of which is associated with a specific whole-brain context. The combination of the localized NC-state and the whole-brain state (outside the network) provides a tractable balance that bridges two analytical levels of cognitive neuroscience: the spatially localized, temporally general network and the temporally localized, spatially general brain state. Thus, our model takes advantage of the spatial simplification provided by canonical networks, which is empirically supported by our findings, while also capturing dynamic reconfigurations of nodes as suggested by dynamic models.

To be sure, this is not the only possible interpretation consistent with our evidence. An alternative model might be developed by first decomposing the whole brain dynamically and afterward identifying spatially localized, synchronous systems that recur over time. Thus, instead of identifying localized connectivity states, this approach would identify transient coalitions of nodes that manifest during particular time windows depending on moment-to-moment affiliations and disaffiliations among nodes. Such coalitions are likely to be, on average, roughly coterminous with network boundaries. While this alternative is consistent with our findings, it is unclear how to identify recurrent coalitions at this time. A developing body of work is beginning to probe this question, for instance using dynamic hypergraph approaches^52, 53^.

Analytically, our framework comprises four main steps, which in principle are extensible to any set of complex brain systems: (i) identify a system of interest (e.g., a canonical brain network), (ii) determine the internal temporal states of that system (here, using dynamic functional connectivity), (iii) determine the external contexts of those internal states (here, either by computing the average connectivity pattern of the whole brain or by identifying co-occurrent states of other systems), (iv) quantify the affiliation between the system’s internal states and external contexts (here, using the WCSS error or the concordance). Notably, our analytical framework provides a better opportunity for capturing multivariate or nonlinear exchanges between networks. This is because our model captures two levels of synchrony dynamics: not only node-to-node connectivity measured within a given temporal window, but also state-to-state concordance. At a particular time, it is possible that network A’s nodes have low moment-to-moment connectivity with nodes in network B, while the states of the two networks at the same time are highly concordant. Consistent with a similar approach in concurrent work^41^, such cases indicate a complex interplay between brain networks that may be reflective of nonlinear or multidimensional interactions among brain regions.

Because fMRI, and dynamic connectivity in particular, is susceptible to a number of spurious phenomena, we took caution to ensure that our results were driven by effects of interest rather than noise. Two artefactual processes are of particular concern in dynamic analyses of resting fMRI: subject motion and sampling variability. First, in-scanner subject motion can bias connectivity results in favour of connections between regions that are close together in physical space^54, 55^. We repeated our experiment on a low-noise subsample of the cohort, which we obtained by censoring epochs of high motion^55^. Overwhelmingly, we observed states nearly identical to those observed in the cohort as a whole, indicating that the dynamic effects we report were not explained by motion. Second, a recent body of work^56^ suggests that transient patterns of brain connectivity are not structured manifestations of a set of underlying brain states; instead, they are artifacts arising due to variable sampling of a single underlying state that is stable across time. To account for this possibility, we generated surrogate data by applying a randomized phase shift to the connectivity data, thus preserving the structure of such a stable underlying state but disrupting any organized dynamic connectivity patterns^56^. On the whole, we observed that connectivity states in the empirical data were better differentiated than those in the surrogate data, indicating that the connectivity patterns we observed reflected multiple underlying brain states. In addition to the analytical precautions taken, our replication of known neural architecture attests to the fidelity of our approach. First, we observed local connectivity states of the default mode network that mirrored previously reported task-related subsystems^15^. Second, our analysis of the independence of local connectivity states recapitulates the known spatial organization of the brain into functional networks^3^. The convergence of dynamic connectivity with previous results from the task-evoked and resting fMRI literature corroborates the argument that dynamic connectivity analyses are capable of detecting true neural architecture and not only spurious fluctuations.

One limitation of the current study is its use of resting fMRI data, which makes inference regarding the functional significance of transient brain states more difficult. Another limitation of the current study is the temporal concatenation of all subject data prior to state detection. While this decision enables a more robust estimation of independence of brain networks across the entire population of subjects and permits us to compute concordances among relatively rare brain states, it leaves the analysis in its presented form poorly suited to examining individual differences in network independence and state concordance features. Current work aims to use task-driven fMRI to afford greater interpretability to the observed network states and to leverage an adapted version of the analysis to detect dynamic network correlates of individual difference variables.

Another potential limitation of the current study is the choice of brain parcellation. We elected to use an ICA-based parcellation because of its ability to identify independent brain regions in a data-driven manner and its allowance of spatial overlap among parcellation nodes. Relative to other approaches (e.g. *a priori* parcellation schemes), ICA-based parcellations offer two comparative advantages: (1) all nodes in an ICA-based parcellation are necessarily independent; and (2) nodes in an *a priori* parcellation may include a mixture of several independent signals. The potential disadvantages of a data-driven parcellation (or any newly introduced parcellation scheme) are primarily in the domains of validity and reliability. In the current study, we established parcellation validity by demonstrating a correspondence between our parcellation nodes and established network architecture. Furthermore, we established parcellation reliability by repeating the ICA procedure using ICASSO, observing convergent results across all repetitions. For these reasons, we believe that an ICA-based parcellation was most appropriate in the current study.

The identification of large-scale brain networks is motivated by the possibility of an improved mapping of brain structure to psychological function. However, the conjectured functions of large-scale brain networks are highly generalized and evade psychological intuition^13^; instead, they reflect the intrinsic degeneracy and pluripotency of the brain^57^. This lack of specificity suggests that brain networks are not the atomic ingredients of neural function. Indeed, analyses of static functional connectivity have revealed that large-scale brain networks are spatially dissociable into subnetworks^50^. Specific subnetworks can be selectively engaged using targeted task conditions^50, 58^, suggesting that they support specific operations of their parent networks’ function. Here, we offer an update to this interpretation using dynamic, time-resolved approaches.

Patterns of neural activity identified as default mode subnetworks are recruited under specific task constraints^50, 58^. We find that the same patterns also occur spontaneously and are detectable in dynamically occurring brain states (Fig. 4). Moreover, our analysis provides additional insights and analytical metrics pertaining to subnetworks from a dynamic framework. First, we provide insight into how nodes – such as those comprising the MTL and DMPFC subsystems of the DMN – behave when one subsystem temporarily dominates over the cohesiveness of the network. In both cases, the non-dominant subsystem appears to demodularize, increasing its connectivity to other networks. Second, whereas time-averaged approaches resolve networks into spatially non-overlapping subnetworks, our analysis extends previous work that predicts spatial overlaps between networks^20–26^. As such, our approach is capable of handling cases of degeneracy and pluripotency, which are considered to an important feature of complex systems^59^. Third, our approach allows us to probe the global context of each subnetwork as reflected in its NC-state (e.g., the between-network connections of the MTL subsystem in DMN2 or DMN3).

Additionally, our concordance analysis (Supplementary Figure 3) shows a remarkably high concordance between SAL2 and EXE2. These NC-states also evidenced subsystems as presented in Fig. 4. Intriguingly, during SAL2, a portion of the salience network, which we termed the ‘cognitive control’ subsystem, joined the executive network (as illustrated in both the chord diagram and the allegiance matrix). In like fashion, during EXE2, a portion of the executive control network joined the salience network. Such findings are consistent with the notion that the network states that correlate with variations in executive function tasks may be reproduced in resting fMRI data^60^. This resemblance between our findings and those from studies using task variables is notable, albeit must be treated with reservation until a direct comparison is made with fMRI data collected during task performance.

According to dynamical systems models, brain activity can be understood as tracing a trajectory through a multidimensional ‘state space’^61^. A question of current interest is how to identify the contributions of different brain regions to the brain’s overall trajectory. Our findings suggest that different types of regions contribute in distinct ways according to their network properties. Specifically, we found that INT nodes (Fig. 6) provide maximal information about the status of the brain as a whole. In that sense, these nodes approximately denote the general location of the brain in its multidimensional state space. Notably, the connections of this system are representative linkages *between networks*. In contrast, the relatively independent *within-network* connections may provide more localized information about the brain’s status, or specific coordinates within its general location (Fig. 5). These findings suggest that the state of the brain is coarsely determined by between-network connections, while within-network connections guide the brain to more specific states.

We also observed that the executive control network was consistently the most independent brain system across validation samples. Prior work has demonstrated that the executive network exhibits a nonspecific or global pattern of connectivity during rest^2, 62^. This nonspecific pattern represents a temporal average over a highly variable dynamic repertoire of connections to all other networks^31, 63^. The independence of the executive control network during rest indicates that its intrinsic activity is relatively unconstrained by activity across the remainder of the cortex. This property of the executive network may enable it to flexibly update its connections and steer the brain into a multitude of difficult-to-access states in response to changing cognitive loads^31, 64^.

The discovery of large-scale functional networks has prompted considerable efforts to understand how these networks relate to individual differences. Prior work has focused primarily on whether canonical networks show topological variation across individuals. However, examining individual differences in network architectures requires a precise characterization of intrinsic connectivity networks. Here our study contributes in several ways. First, extending previous work in dynamic connectivity^32, 35, 36^, our findings suggest that canonical network analyses of individual differences run the risk of conflating inter-subject differences in state topology with differences in network topology. Because each network can be resolved into distinct NC-states, it may be more informative to isolate states whose between-subject connectivity differences most strongly relate to individual difference variables. Second, our findings introduce novel approaches for relating differences between individuals to differences in network architectures. Dwell times^35^ and transition frequencies^65^ of whole-brain states have been identified as correlates of schizophrenia; analogous metrics computed for localized states could elucidate network drivers of pathology. Furthermore, the contextual independence metrics that we introduce might illuminate previously overlooked correlates of individual difference variables; specific pathologies may be reflected in a failure of systems to coordinate or, reciprocally, a failure of systems to segregate (elevated independence or interdependence). While the application of our approach to individual differences research is left to future work, the methods we present for examining the degree of independence of brain systems could illuminate new relationships.

Researchers have long acknowledged that brain networks are not immutable, monolithic entities, but analytical strategies consistent with this acknowledgment have been difficult to reconcile with the extensive corpus of static network neuroscience literature. Methods aimed at recapitulating canonical networks fail to capture important dynamics occurring within and between those networks. However, analyses that do away with network assumptions often present challenges of interpretability and complexity. Our approach, *contextual connectivity*, addresses this issue by introducing an intermediate level of analysis that not only respects the robust finding that networks are relatively autonomous, but also recognizes that networks are at best superordinate approximations of dynamically recurring states. As such, NC-states provide a tractable approximation of the functional connectome that maintains fidelity across both spatial and temporal levels of analysis, and thus may be valuable for examining relationships between networks and dynamic whole-brain architectures.

## Methods

### Subjects

Minimally preprocessed resting fMRI data for 200 healthy adult humans (age 22–35, 112 female) were selected randomly from the Human Connectome Project S500 public data release^66^. We selected 200 participants in order to perform a split-half reliability test on subsamples of 100 subjects each. Samples of similar (or smaller) size have previously been used to successfully define and identify functional brain networks^2, 46^ and to perform dynamic connectivity analysis^25, 36^. Acquisition of data received institutional review board approval from the Washington University institutional review board; data analysis was approved by the Human Subjects Protections Committee (Institutional Review Board) at Pomona College. Informed consent was obtained in accordance with the policies of the host institution, and data were de-identified prior to analysis. All methods were performed in accordance with the relevant ethical guidelines.

### Image acquisition and preprocessing

Data were acquired on the 3T Connectom scanner (Siemens Healthcare, Erlangen, Germany) using multiband pulse sequences (*T*_*R*_ = 720 ms; *T*_*E*_ = 33.1 ms; 2.0 mm isotropic spatial resolution; multiband factor = 8)^67–70^. During resting data acquisition, subjects were instructed to visually fixate on a crosshair. The data acquisition strategy is detailed elsewhere^71^.

Data were obtained as outputs of the Human Connectome Project’s denoising pipelines, detailed elsewhere^72^. In addition to standard fMRI preprocessing using FSL and FreeSurfer^73, 74^, data were denoised to minimize the impact of subject movement on connectivity estimates. In brief, subject data were decomposed using ICA, and nuisance signals were removed via regression of realignment parameters, their temporal derivatives, and independent components identified as artifactual by a trained classifier (ICA-FIX^75, 76^).

In order to divide the brain into functional parcels, we used group-level independent component analysis (1) to decompose the preprocessed images into 100 constituent signal sources common to all 200 subjects and (2) to identify where in the brain each signal was localized^42^. Following previous work^32, 77^, we selected a relatively high model order (100 components) in order to obtain a fine-grained parcellation with components corresponding to functional and anatomical units of brain organization. We then applied back-reconstruction (GICA1) to obtain, for each subject, 100 spatial maps and timeseries representing subject-specific analogues of each group-level independent component^78, 79^. Component validity was assessed both qualitatively (via visual inspection) and via cross-correlation of component maps with canonical network maps^46^; components corresponding to movement or physiological noise were discarded, and 80 of the original 100 components were retained as functional parcels, or network nodes. Although network nodes are sometimes constrained by a criterion of spatial contiguity (e.g., *a priori* ROIs), ICA decomposition does not enforce this criterion. Because we used a high-dimensional ICA decomposition of the data, however, the independent components that we used as network nodes were, for the most part, localised to specific brain regions. Following conventions from previous work that used a similar approach^25, 32, 35, 36^, nodes were named according to the spatial localisation of their peak coordinates in combination with a qualitative visual inspection of component maps. The activation timeseries of each node was subject to additional preprocessing steps in the GIFT toolbox, including demeaning and detrending, interpolation over artifact-related outliers (‘despiking’), removal of frequencies less than 0.01 Hz or greater than 0.15 Hz using a bandpass filter, and variance normalization of signal intensities.

### Canonical network discovery

The time-averaged functional connectivity between each pair of processed node timeseries was computed as the Pearson correlation coefficient^80^. This analysis yielded a symmetric, undirected graph with 3160 edges. The weight of the edge connecting node *n*_*i*_ to node *n*_*j*_ was encoded as feature *E*_*ij*_ in a symmetric 80 × 80 adjacency matrix *E*. This time-averaged connectivity matrix was used to separate nodes into canonical networks and to establish a reference against which transient connectivity metrics could be compared.

We applied a generalized Louvain-like community detection algorithm^44, 45^ to a 70 × 70 submatrix of this adjacency matrix; this submatrix corresponded to cortical nodes and their connections. The community detection algorithm that we used (one of the most widely used community detection algorithms in network neurosciences) requires specification of a resolution parameter. The value of this resolution parameter determines the number of networks into which the brain is subdivided, as well as the spatial extent of these networks. In order to select an appropriate resolution parameter, we required a hypothesis that described the number and spatial extent of canonical brain networks in the neuroscience literature, so that we could choose the resolution parameter that most closely approximated this hypothesis. The hypothesis that we used was based on an *a priori* partition of the cerebral cortex, in which canonical brain networks are typically anchored^3^. Thus, because the hypothesis only provided information about cortical networks, community detection was restricted to a 70 × 70 submatrix consisting only of cortical nodes. The Louvain resolution parameter was trained by performing community detection at a number of resolutions and penalizing the distance between the resultant partition and an established *a priori* partition^3^ (Supplementary Figure 1) using the following cost function. The cost function was computed based on the community assignments for each pair of nodes *x* and *y*, and then the sum over all unique pairs was used as the overall value of the cost function:

$$x\cdot y=\{\begin{array}{ll}0 & x=0,\,y=0\\ \frac{n}{m-n} & x=0,\,y=1\\ 1 & x=1,\,y=0\\ -1 & x=1,\,y=1\end{array}$$

wherein for each pairwise connection,

*x* represents the generalized Louvain partition’s prediction about the community status of the pairwise connection;

*y* represents the hypothesis partition’s prediction about the community status of the pairwise connection;

*x* is 1 if the Louvain algorithm for the thresholded adjacency matrix partitions the network such that both elements in the pair are in the same community;

*y* is 1 if the hypothetical-spatial model predicts that both elements of the pair are in the same community;

*x* is 0 if the Louvain algorithm for the thresholded adjacency matrix partitions the network such that the elements of the pair are in different communities;

*y* is 0 if the hypothetical-spatial model predicts that the elements of the pair are in different communities;

*m* is the number of intramodular pairwise connections in the hypothetical-spatial model; *n* is the total number of nodes in the network.

Because the *a priori* network partition used to train the algorithm included only the cerebral cortex, we limited the scope of the community detection algorithm to cortical nodes. Using this approach, we partitioned cortical nodes into six canonical networks. The 10 subcortical nodes were then assigned to a seventh subsystem.

### Dynamic functional connectivity and NC-state resolution

Dynamic functional connectivity among the 80 nodes was computed over a 44.64 s tapered (rectangle convolved with a Gaussian) sliding window incremented 0.72 s over 14.4-minute node timeseries^81^. In the absence of information about the timescale of dynamic fluctuations in connectivity, the probability of detecting such fluctuations in resting fMRI data is optimized for a sliding window approximately 50 s in duration^56^. Because the data included more features than observations, the pairwise connectivity matrix during each time window was computed as a regularized precision matrix^32, 82–84^. An aggressively denoised subsample of all data was selected by computing the mean framewise displacement^55^ during each time window and excluding any windows with a mean FD > 0.18 mm; NC-state identification (as described below) was performed on the full sample and this subsample with comparable results. Whereas previous work^55^ used a framewise displacement threshold of 0.2 mm as a criterion for censoring, we elected to use a more conservative criterion because framewise displacement averaged over a 44.64 s window tends to smooth over single-volume outliers. We selected a value of 0.18 mm so that approximately one-third of windows were censored.

We generated six network-specific graphs for each time window by extracting from the whole-brain graph only edges between nodes in the same network. Following an approach previously used to detect connectivity states^32^, we used k-means clustering (L1 distance) of these window-wise graphs to identify time windows during which each network exhibited relatively consistent connectivity patterns. We determined the number of clusters (connectivity patterns) for each network using a semi-formalized elbow criterion (Supplementary Figure 2). To ensure the validity of clustering, we performed clustering on data generated by independently permuting observations of each variable in order to preserve variable distributions without maintaining any explicit relationship between the variables. We found that the sum-of-squares error for the null data significantly exceeded that for the observed data (*p* < 0.01), a positive indication that a clustering approach was valid (Supplementary Figure 2C). We thus obtained for each network a set of cluster centroids along with a map assigning each time window to a centroid. We defined each centroid as a NC-state, or connectivity state. To ensure that NC-states represented dynamic reconfiguration within subjects rather than individual differences across subjects, the number of NC-states represented in each subject was computed. We also repeated connectivity state detection, as above, using all nodes in the cerebral cortex rather than only those assigned to a particular network.

To validate results, the clustering procedure was repeated on randomly selected split-half samples of the data. Similarity of subsample centroids was then assessed using the correlation distance metric (1 − *r*, where *r* is the pairwise Pearson correlation between the connections of subsample centroids) as a proxy for similarity. In addition to the empirical split-half subsamples, clustering was performed on surrogate data (permuted split-half samples) generated by applying a random phase shift to the timeseries representing the strength of each connection within the network of interest over time. The similarity between empirical split-half centroids and analogous phase-shifted split-half centroids was then assessed using the correlation distance metric. Each centroid was reported as replicated if clustering of the empirical split-half samples produced centroids more similar to one another than to the centroids yielded by clustering the phase-shifted data. Each centroid was reported as replicated if it satisfied both of two criteria: first, similarity across split-half samples and second, greater-than-chance similarity across split-half samples. To determine the overall similarity of centroids across split-half samples, we randomly divided our data into two equal subsamples, then performed clustering separately on both subsamples. Each centroid was considered replicable on the basis of the feature-wise correlation coefficient between that centroid in the first split-half sample and the closest centroid in the second split-half sample (replicable if r > 0.8). To determine whether similarity of centroids was greater than chance, we applied a phase randomization to all dynamic edge timeseries, and then performed clustering on the phase-randomized split-half samples 1000 times for each network. Finally, we examined whether the similarity of centroids computed using the real data was significantly greater than the similarity of centroids computed using the surrogate data.

When the first criterion (similarity >0.8) was applied, 7 states (VIS3, VIS4, SOM3, DAT3, DAT7, DMN5, and DMN8) did not replicate. When the second criterion (similarity greater than chance) was applied, 13 states (VIS1, VIS3, SOM2, SOM3, SOM8, DAT3, DAT7, DAT8, SAL8, DMN1, DMN4, DMN5, and DMN8) did not replicate. A total 6 states (VIS3, SOM3, DAT3, DAT7, DMN5, and DMN8) were flagged by both approaches; in general, testing whether split-half states were more similar than chance proved to be a stronger criterion. The majority of states (32) were not flagged by either criterion. Qualitative visual inspection of all subsample centroids suggested replication rates similar to but greater than this automated approach.

### Contextual connectivity and concordance

For each subject, we used Louvain-like multilayer community detection to compute each node’s community membership at every point in time^45^. For each NC-state, we identified an average whole-brain connectivity context (WBCC) by computing a community-allegiance matrix over all time points in which the network exhibited the NC-state in question. In the allegiance matrix, an edge *E*_*ij*_ connecting nodes *n*_*i*_ and *n*_*j*_ is assigned a weight equal to the probability that *n*_*i*_ and *n*_*j*_ are assigned to the same community over the sampled time points (the “allegiance” of those nodes to one another)^85^. These context-specific allegiances were next quantified as a ‘displacement from baseline allegiance’, defined as the ratio of within- and between-network allegiances in a specific WBCC to the same within- and between-network allegiances averaged over all time. To facilitate visualization, these ratios were rescaled to represent relative shares of total allegiance in each WBCC and plotted on an exponential scale.

Additionally, a Bayesian ‘concordance’ metric was computed, which indexed the change in the probability that a particular network (or the whole cortex) exhibited a particular NC-state (or connectivity state) given information about the NC-state (or connectivity state) of another network (or the whole cortex):

1 $$C({A}_{i},{B}_{j})=ln(\frac{P({A}_{i}|{B}_{j})}{P({A}_{i})})$$

wherein for each pair of network-specific NC-states,

*A*_*i*_ represents NC-state *i* of network *A*;

*B*_*j*_ represents NC-state *j* of network *B*, where *j* may equal *i*.

Concordance was zero-centred by applying a logarithm to the posterior-to-prior probability ratio; positive concordances thus corresponded to states more likely to co-occur than predicted by chance, while negative concordances corresponded to states less likely to co-occur than predicted by chance.

Three null models were used to generate control distributions of concordance data under the assumption of complete independence of the networks. These were generated by shuffling or simulating the assignment of each time point to a particular set of NC-states. In the first model, the observed NC-state assignments for each network were randomly permuted across subjects. In this way, the observed trends of occurrence of connectivity states over time was preserved, but any explicit relationship between NC-states in different networks was abolished. In the second model, NC-state assignments were simulated using the observed initial conditions and Markov chain transition models computed from the observed data. In the third model, the phase of the observed NC-state assignments was randomly shifted. In this way, any apparent concordance that was attributable to static individual differences (or to sampling variability) was preserved, but dynamic concordances were abolished. A concordance matrix was then generated, as above, for the permuted or simulated data. Null distributions for hypothesis testing were generated from 1000 repetitions of each null model. If a concordance was non-significant under any of the three null models, then it was marked as non-significant.

### Subsystem identification

Four NC-states (DMN2, DMN3, EXE2, and SAL2) corresponding to fractionation of a brain network into subsystems were initially identified qualitatively. Dynamically engaged subsystems of brain networks were then identified through hierarchical clustering of the average connectivity profiles of all nodes in these NC-states (Supplementary Figure 4). Hierarchical clustering was performed using the correlation distance metric, defined as 1 − *r*, where *r* is the Pearson correlation coefficient between the compared connectivity profiles. Subsystems of brain networks were considered to be recruited in a particular NC-state if the correlation distance between the connectivity profiles of their constituent nodes did not exceed 0.4. Our objective in selecting this distance threshold was to consistently identify the largest coherent subsets of nodes across the four NC-states without capturing trivial subsystems (2 coupled nodes). A correlation distance cutoff of 0.4 yielded, across all networks, no such trivial systems consisting of 2 nodes. Varying the threshold between 0.4 and 0.6 produced some differences in node assignments, but did not affect the overall association of nodes. Smaller and greater thresholds resulted in poorly interpretable subsystems consisting only of paired nodes.

### Evaluation of null/independence hypothesis

We evaluated the null hypothesis of complete network independence by comparing the empirical contexts of each NC-state to phase-randomized contexts of each NC-state. For each network, we applied a randomized phase shift to the timeseries representing the strength of each connection over time^32, 56^ (the edge-weight timeseries). Only connections outside of the network of interest were phase-shifted in this manner; thus, dynamic structure was preserved within each network but abolished for the remainder of the brain. Contexts were then computed for each NC-state for the phase-randomized data, as described above.

Our analysis was predicated upon the following assumptions:

- Randomly phase-shifting each edge-weight timeseries preserves the mean and variance of the timeseries, but disrupts the overall dynamic covariance structure^32^, which is dependent upon common features across multiple edge-weight timeseries.
- If a network’s intrinsic connectivity state were independent of its whole-brain environment, then the network’s states would not co-occur with any consistent changes in the covariance structure of the remainder of the brain.
- If networks are completely independent, then a randomized phase shift of all edge-weight timeseries not in the network of interest will not meaningfully change the similarity between contexts.

When we applied a randomized phase-shift to all edge weight timeseries outside of a network of interest, we instead observed that the resultant phase-shifted NC-state contexts were, without exception, more similar to one another than were the empirical contexts of NC-states (*p* ≪ 0.001, paired Wilcoxon signed-rank test), suggesting that NC-states occurred in the context of specific changes in the whole-brain connectivity structure.

### Independence

The independence of each canonical network was computed using two metrics: mutual variation and mean concordance. For each network, null distributions for each metric were generated on the basis of 1000 *pseudo-networks*. Each pseudo-network was defined to include the same number of nodes and NC-states as the canonical network in question. However, unlike the case for canonical networks, nodes were assigned to pseudo-networks randomly and not on the basis of community structure or previous scientific results. Pseudo-network NC-states were then computed using k-means clustering in a manner analogous to canonical network NC-states. For each canonical and pseudo-network NC-state, contexts were obtained (1) as allegiance matrices, defined as the probability that each pair of nodes would be assigned to the same community while that particular NC-state was present, and (2) as contextual centroids, defined as the mean of all Fisher-transformed whole-cortex windows during which a network or pseudo-network expressed that NC-state.

For independence analysis using the mutual variation metric, contextual centroids were treated as a proposed clustering solution for the entire cortex, with the understanding that a network that was less independent of the whole cortex would provide a better clustering solution for the cortex. The goodness of this clustering solution was thus computed as a proxy for the network’s independence from the cortex as follows. The correlation distance from each z-transformed whole-cortex window to the nearest contextual centroid was computed (the “within-cluster” distance). All distances were squared and subsequently added together to determine a within-cluster sum-of-squares (WCSS) error term. The WCSS reflected the extent to which the proposed clustering solution (which was based only on information about the temporal variance of a single canonical or pseudo-network) explained the temporal variance present in the entire cortex; a lower error term corresponded to a better clustering solution and thus to less independence.

The theoretical upper limit on clustering efficacy (maximal interdependence) corresponded to the clustering solution that minimized the WCSS error; this was obtained by clustering all cortical features (taking into account the temporal variance of the entire cortex rather than only that of its subsystems). The theoretical lower limit on cluster efficacy (maximal independence) would result in maximization of the WCSS error and corresponded to centroids identical to the time-averaged cortical connectivity except in the network of interest, where they were identical to the network’s NC-state centroids. Permutation tests for significance were performed for each canonical network’s WCSS error score relative to the null distribution generated from the 1000 pseudo-networks with similar properties. Following the independence analysis, an independence score was obtained for each network by scaling the mean WCSS error among pseudo-networks to zero, maximal interdependence to −1, and maximal independence to 1.

A second assay for independence was performed using the concordance metric. Here, interdependence was operationalized as the concordance of (pseudo-)network NC-states with 8 whole-cortex states, with positive and negative concordances computed separately because of their different interpretations. More interdependent systems were predicted to exhibit greater positive and negative concordances. The mean positive and negative concordances between (pseudo-)network NC-states and whole-cortex states were separately computed. Results using this metric (with the exception of those for the somatomotor network) were convergent with the results from the WCSS approach.

### Identification of a highly interdependent system

The “interdependent” (INT) system was identified through an exploratory two-step process. First, a tally was obtained of the frequency with which nodes appeared in pseudo-networks that scored in the bottom quintile of WCSS errors. A subset of nodes that frequently often occurred in such “interdependent” networks was thus identified, and pseudo-network generation (8 nodes, 8 NC-states) was repeated 1000 times, with random drawing from only this subset of nodes. On the whole, the resultant pseudo-networks exhibited notably lower WCSS errors than did those selected from all nodes. Among these pseudo-networks, the one with greatest interdependence (lowest WCSS error) was selected as the INT system. Interdependence was re-evaluated and reproduced in split-half samples and with 16 NC-states and cortical states instead of 8. Independence was also evaluated for pseudo-networks generated from nodes with (1) maximal participation coefficient and (2) mean allegiance to nodes other than canonical partners without significant results.

## Electronic supplementary material

Supplementary Information
