# Supplementary material for: Contextual connectivity: A framework for understanding the intrinsic dynamic architecture of large-scale functional brain networks
Source: Sci Rep. 2017 Jul 26;7:6537. doi: 10.1038/s41598-017-06866-w (PMC5529582; doi:10.1038/s41598-017-06866-w)
Supplement: Supplementary file 1 — Supplementary Information [file 41598_2017_6866_MOESM1_ESM.pdf]

# **Supplementary Information** | Contextual connectivity: A framework for understanding the intrinsic dynamic architecture of large-scale functional brain networks

Rastko Ciric, Jason S. Nomi, Lucina Q. Uddin, Ajay B. Satpute

## Supplementary Figures

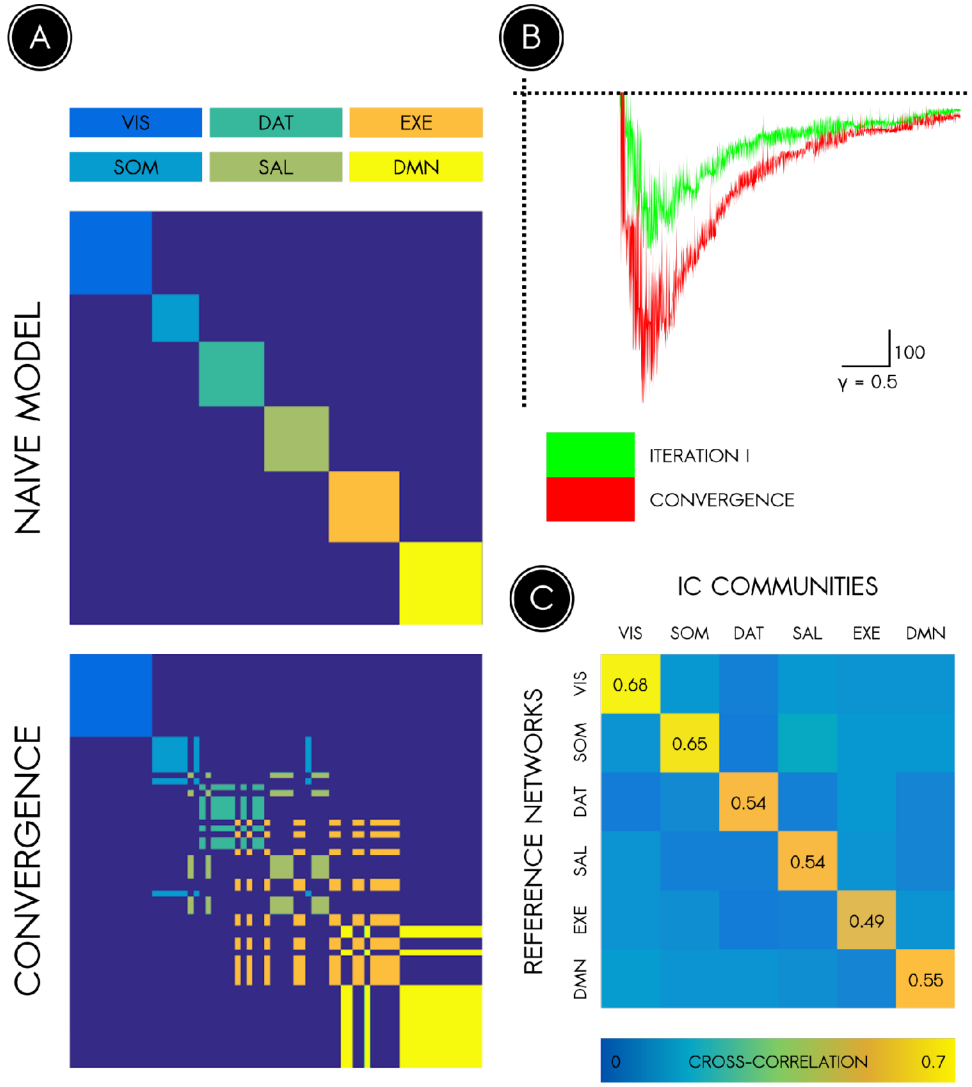


**Supplementary Figure 1 | Training the resolution parameter of the generalized Louvain algorithm to discover canonical networks.** An existing 7-network partition of the cerebral cortex (Yeo et al., 2011) was used to train the resolution parameter of the generalized Louvain algorithm. **(A)** *Top*, before community detection was performed, each network node was assigned to one of 6 *a priori* networks by cross-correlating the node's spatial map with the spatial maps of all *a priori* networks, producing a naive model based on spatial information alone. In the visualization of the adjacency matrix, within-network edges are color-coded (key, top left), while between-network edges are dark blue. This naive partition served as a model for training the community detection algorithm. *Bottom*, the converged solution upon applying the community detection algorithm is similar to the naive spatial model above but also refines it so as to respect the connectivity structure in the resting data, as illustrated by discrepancies from the naive model. **(B)** The community detection algorithm requires tuning a resolution parameter (gamma, abscissa, scale on lower right), which determines the number and spatial extent of networks in the resting data. We defined a cost function (ordinate) to represent the distance between the model partition and the partition estimated by the community detection algorithm (see Methods). The first iteration is shown in green for comparison with the converged solution (red). The optimal solution consistently occurred at a value of gamma near 1.3. We then updated the model partition by cross-correlating the combined maps of each Louvain community with the maps of *a priori* networks. We repeated this process of community detection and updating the model target partition until the evidence from community detection converged (i.e. A, bottom). **(C)** Spatial cross-correlations of communities of nodes with reference networks from the *a priori* partition ranged from approximately 0.5 to 0.7, establishing a one-to-one correspondence between our communities and the canonical reference networks.

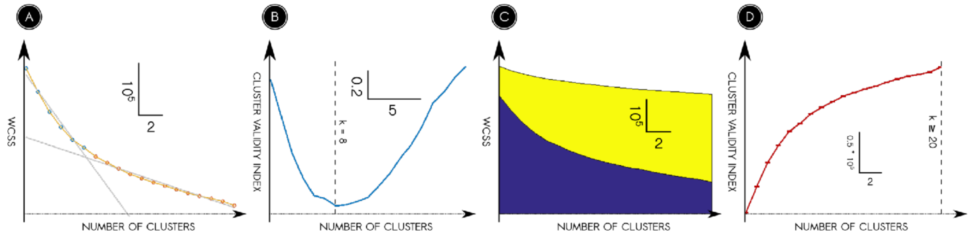


**Supplementary Figure 2 | Validity of clustering-based data reduction is established via permutation. (A)** One challenge inherent in the k-means approach is selecting the number of clusters, or NC-states. In order to determine the number of NC-states for each network, we applied a semi-formalized 'elbow criterion'. An example cluster determination plot for the salience network illustrates the elbow computation for the optimal number of clusters (8). The number of clusters is plotted on the abscissa, while the variance explained (within-cluster sum of squares criterion) is plotted on the ordinate. The putative number of clusters was varied from 2 to 20. Two least-squares lines were fit to the resultant plot, with the putative number of clusters demarcating the point of separation between data included in each computation. The putative solution that optimized fit for both lines was selected as the number of clusters. **(B)** A plot of the cost function for the elbow criterion, as determined by subtracting from 1 the product of the correlation coefficients of the two lines from (A). The semi-formalized elbow criterion suggested an optimal solution of $k = 8$ clusters. **(C)** To ensure that a clustering approach was valid in these data, we performed a permutation test that preserved the distributions of all variables, but abolished the relationships between them that would have encoded any cluster structure. The empirical clustering validity plot for the salience network (blue) is plotted for comparison with the mean null clustering validity plot (yellow) across a range of $k$. **(D)** A more formalized gap criterion suggested an optimal solution of at least 20 clusters. Though more quantitatively rigorous, this approach was not used because many of the resultant NC-states would have been idiosyncratic (present in only a single subject). Error bars indicate standard deviation.

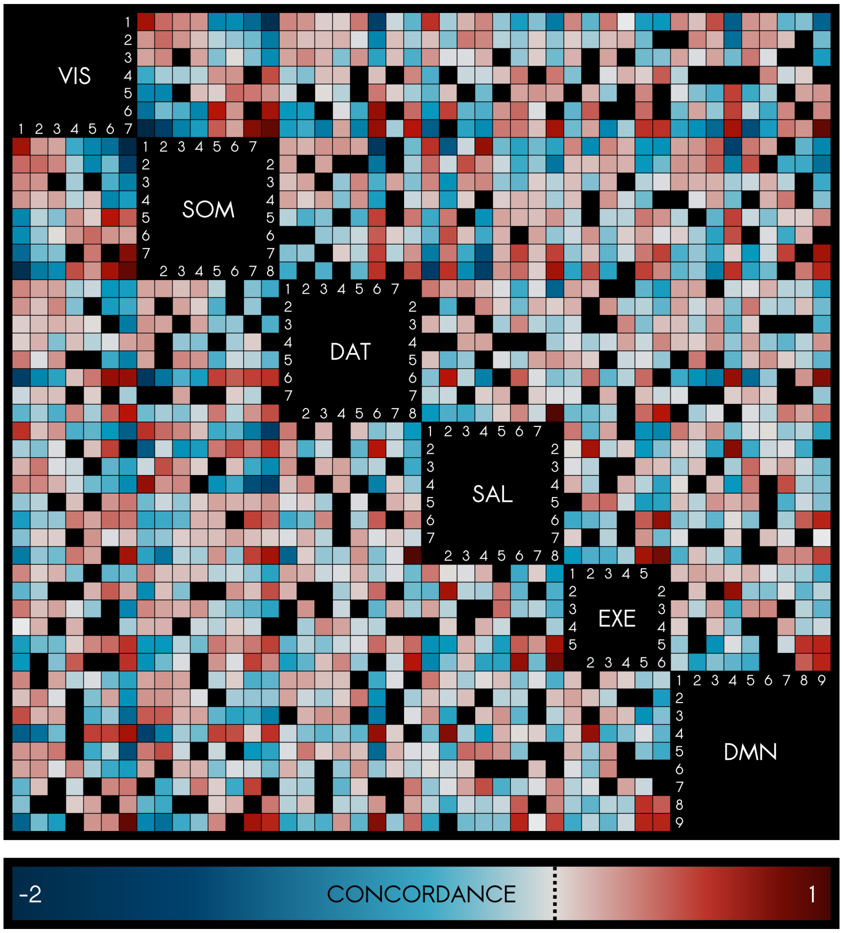


**Supplementary Figure 3 | Concordance matrix across NC-states: Functional interdependence of networks..** The figure illustrates pervasive interdependence between canonical networks when examined at the level of NC-states. In comparison with the univariate region-to-region functional connectivity used in many neuroimaging studies, our approach leverages dynamic tools to enable a multivariate state-by-state estimation of network independence. Cooperativity between brain systems is revealed in a state-by-state matrix of Bayesian concordances, which captures co-occurrences of single-system connectivity patterns that deviate from the prior probability derived under a model of local-global independence of brain systems. Compared with permuted and simulated null models assuming independence, nearly all NC-states exhibited a significant degree of concordance or discordance ($p < 0.05$, Bonferroni corrected). The fewer non-significant concordances are blacked out for ease of visualization.

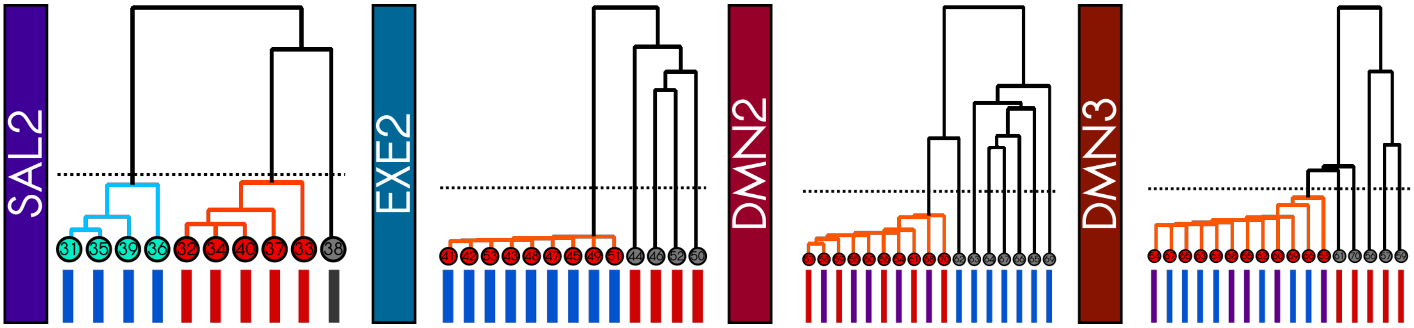


**Supplementary Figure 4 | Criterion for subnetwork identification.** Qualitative inspection suggested that the salience, executive, and default mode networks exhibited states reflective of fractionation or bifurcation into subnetworks. To identify dynamic subnetworks, the connections of each of those networks' nodes were clustered hierarchically. The resultant dendrograms are shown here for the states displayed in Figure 4. A hard cut-off at a correlation distance of 0.4 was used to define cohesive subnetworks. The circular dendrogram leaves are colour-coded according to the cluster assignment of each node; the vertical bars underneath the leaves are colour-coded according to subnetwork assignment. For the default mode network, red bars correspond to the MTL subsystem (assigned to a cluster in DMN2 but not DMN3), blue bars correspond to the DMPFC subsystem (assigned to a cluster in DMN3 but not DMN2), and violet bars correspond to the midline core subsystem (assigned to a cluster in both NC-states). While not cohesive, nodes of the auxiliary executive subsystem did display some common changes in overall connectivity.
